# Supplementary material for: Engineering water exchange is a safe and effective method for magnetic resonance imaging in diverse cell types
Source: J Biol Eng. 2024 Apr 22;18:30. doi: 10.1186/s13036-024-00424-5 (PMC11035135; doi:10.1186/s13036-024-00424-5)
Supplement: Supplementary file 1 — Supplementary Material 1 [file 13036_2024_424_MOESM1_ESM.docx]

**Engineering water exchange is a safe and effective method for magnetic resonance imaging in diverse cell types**

Austin D.C. Miller^1^, Soham P. Chowdhury^2^, Hadley W. Hanson^1^, Sarah K. Linderman^2^, Hannah I. Ghasemi^2^, Wyatt D. Miller^1^, Meghan A. Morrissey^2^, Chris D. Richardson^2^, Brooke M. Gardner^2^, Arnab Mukherjee^1,3,4,5,6^

Affiliations:

^1^Biomolecular Science and Engineering Graduate Program, ^2^Department of Molecular, Cellular, and Developmental Biology, ^3^Department of Chemical Engineering, ^4^Department of Bioengineering, ^5^Department of Chemistry, ^6^Neuroscience Research Institute, University of California, Santa Barbara, CA 93106, USA

*Correspondence should be addressed to AM (arnabm@ucsb.edu)

**Supporting Figures**

**
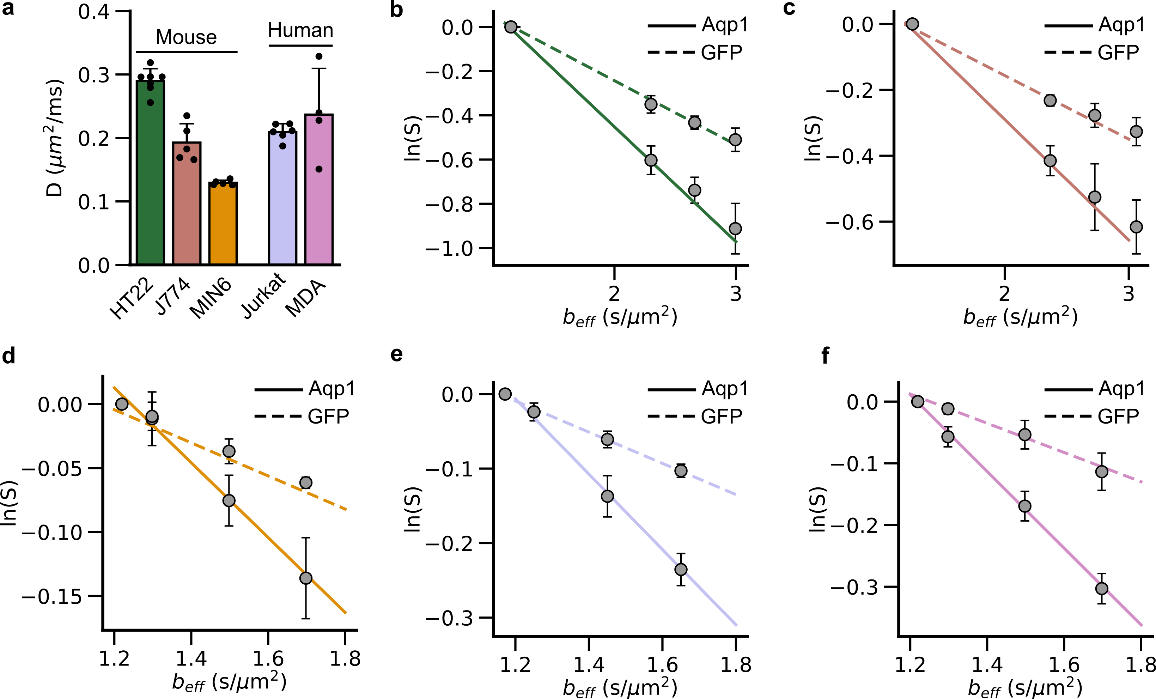
**

**Figure S1: Measurement of diffusivities in wild-type and Aqp1-expressing cells**. **a**, Baseline diffusivities were measured in cells transduced to express GFP. Aqp1 expression increases diffusivity over baseline, leading to a faster decay in diffusion-weighted signal intensity ($S$) with effective b-value ($b_{eff}$) in all five cell types: **b**, HT22, **c**, J774, **d**, MIN6, **e**, Jurkat, and **f**, MDA-MB-231 cells. The solid and dotted lines represent the first-order decay in signal intensity in Aqp1- and GFP-expressing cells, respectively. Error bars represent s.e.m. (*n* ≥ 4 biological replicates). All MRI data were acquired at 7 T, using a diffusion time of 300 ms.


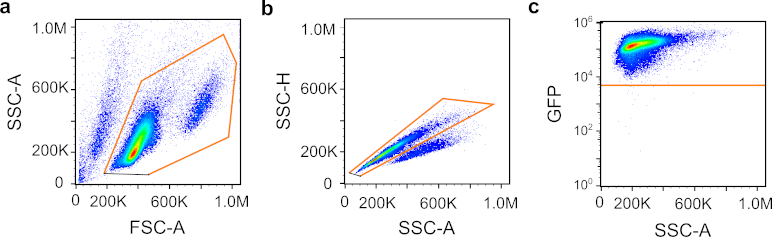


**Figure S2: Gating strategy to select reporter-expressing following lentiviral transduction**. Representative flow cytometry analysis depicting the gating strategy used to generate uniform populations of reporter-expressing cells by gating for **a**, viability **b**, singlet cells, and **c**, GFP expression. FSC-A indicates forward scatter area. SSC-H and SSC-A respectively denote side scatter height and side scatter area.


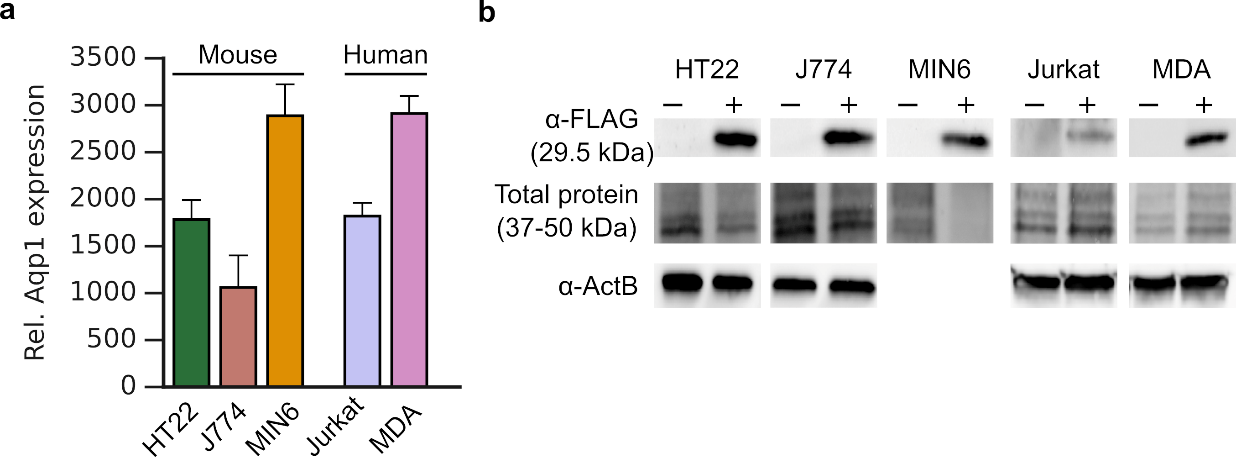


**Figure S3: Detection of Aqp1 transgene expression**. **a**, Expression of the Aqp1 transgene was detected by qRT-PCR using a forward primer that binds to the FLAG epitope sequence incorporated at the N-terminus of Aqp1. GAPDH and actin were used as housekeeping genes for the mouse and human cell lines, respectively. Relative Aqp1 expression was quantified using the $2^{-\Delta\Delta C_{t}}$ method. **b**, Immunoblotting was used to detect the expression of Aqp1 protein in whole-cell lysates, utilizing anti-FLAG antibodies. As a loading control, the total protein in each lane was visualized by UV illumination, employing a stain-free technique. The use of β-actin (ActB) served as an additional loading control, with the exception of the MIN6 cell line where the entire lysate was utilized for Aqp1 detection. Aqp1- and GFP-expressing (control) cell lines are indicated by ‘+’ and ‘-’ respectively. Error bars in (a) represent the s.e.m. from *n* = 3 biological replicates.

**
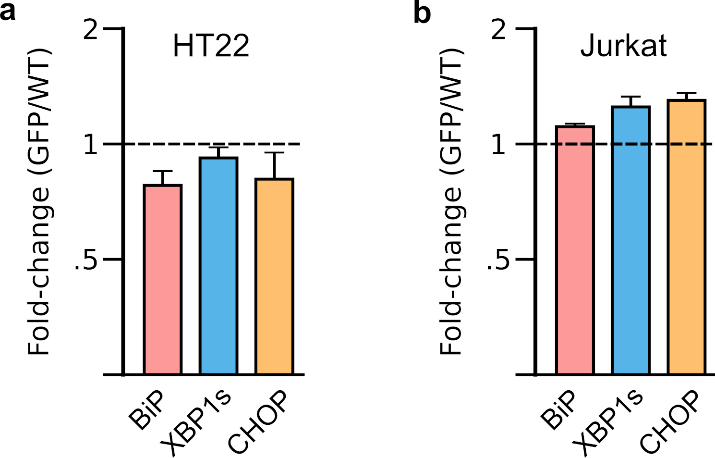
**

**Figure S4: Effect of GFP expression on the unfolded protein response**. Fold changes in key UPR-associated genes, BiP, XBP1s, and CHOP in GFP-expressing cells relative to wild-type controls in mouse (HT22) and human (Jurkat) cell-lines. GAPDH and actin were used as housekeeping genes for the mouse and human cell lines, respectively.


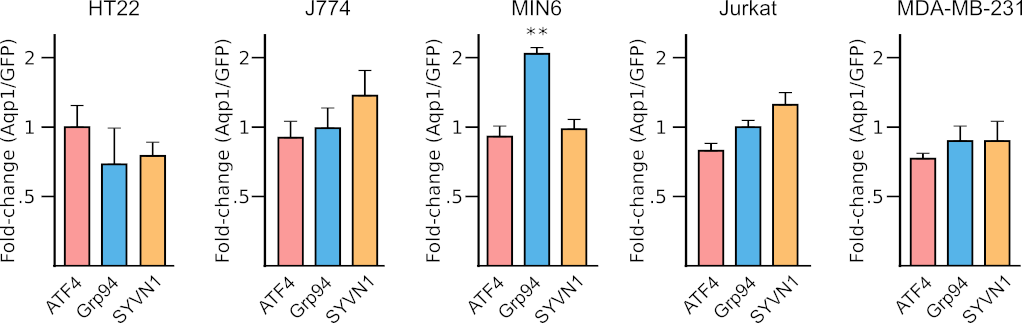


**Figure S5: Effect of Aqp1 expression on the unfolded protein response**. Fold changes in the expression of UPR-associated genes, including ATF4, Grp94, and SYVN1 in Aqp1-expressing cells relative to GFP controls. Error bars represent the s.e.m. from *n* = 3 biological replicates. ** *P*-value < 0.01 (2-sided t-test). GAPDH and actin were used as housekeeping genes for the mouse and human cell lines, respectively.

**
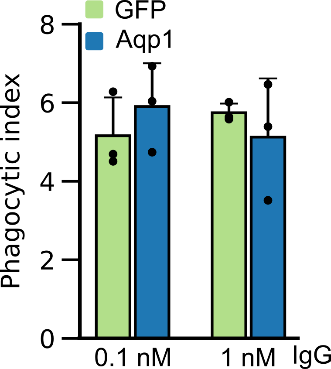
**

**Figure S6: Phagocytic activity of Aqp1- and GFP-expressing macrophage cells**. Phagocytic index was computed following exposure of J774 cells to lipid-coated silica beads with 0.1 or 1 nM IgG1κ, for 45 min.


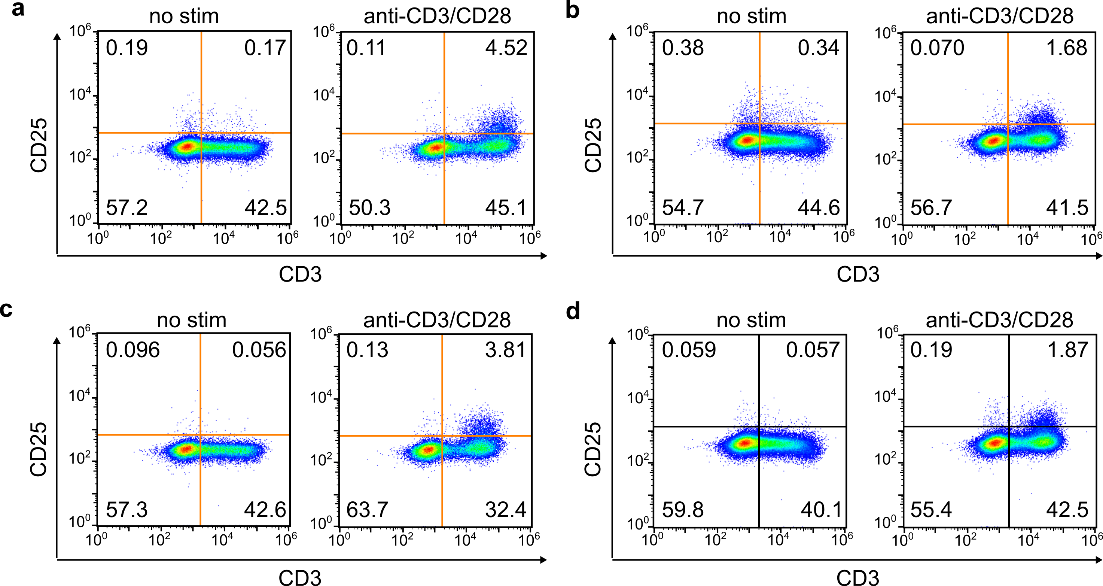


**Figure S7. Flow cytometry analysis of CD25 and CD3 surface expression in Jurkat cells.** To stimulate CD25 expression, Jurkat cells were incubated with anti-human CD3/CD28 beads at a 5:1 bead-to-cell ratio for 24 h. (**a,c**) Aqp1- and (**b,d**) GFP-expressing Jurkat cells were analyzed for surface expression of CD3 (pan T-cell marker) and CD25 (activation marker) using flow cytometry. The numbers in each quadrant denote the fraction of cells showing surface expression of CD3, CD25, both CD3 and CD25, or neither marker.

| **Table S1**: General characteristics and in vivo relevance of cell lines used in the study | | | | | |
| --- | --- | --- | --- | --- | --- |
| **Cell line** | **Type** | **Species** | **Sex** | **Culturing** | **Potential in vivo relevance for Aqp1 reporter gene imaging** |
| HT22 | hippocampal neuron | mouse | unknown | adherent | Mapping neural gene expression at the whole-brain scale in small and large animal models |
| J774A.1 | macrophage, monocyte | mouse | female | adherent | Cell-tracking in animal models of cell-based immunotherapy |
| MIN6 | insulinoma, pancreatic beta cell | mouse | unknown | adherent | Imaging pancreatic function in disease models (e.g. diabetes) and cell-based therapies (e.g., islet cell transplants) |
| Jurkat E6-1 | T lymphocyte | human | male | suspension | Cell-tracking in animal models of cell-based immunotherapy |
| MDA-MB-231 | epithelial, triple negative breast cancer | human | female | adherent | Longitudinal tracking of viable tumor cells, metastatic gene expression (e.g., cancer stem cells), tumor microenvironment (e.g., hypoxia), and therapeutic gene and oncolytic viral delivery |

| **Table S2**: Gene sequence and oligonucleotide primers for qRT-PCR | |
| --- | --- |
| **Primers/gene** | **Sequence** |
| Aqp1 (the FLAG epitope sequence is shown in bold; binding sites for forward and reverse primers, viz., Aqp1_F and Aqp1_R are underlined) | atg**gactacaaggacgacgacgacaag**gccagcgagttcaagaagaagctcttctggagggcagtggtggccgagttcctggccacgaccctctttgtcttcatcagcatcggttctgccctgggcttcaaatacccggtggggaacaaccagacggcggtccaggacaacgtgaaggtgtcgctggccttcgggctgagcatcgccacgctggcgcagagtgtgggccacatcagcggcgcccacctcaacccggctgtcacactggggctgctgctcagctgccagatcagcatcttccgtgccctcatgtacatcatcgcccagtgcgtgggggccatcgtcgccaccgccatcctctcaggcatcacctcctccctgactgggaactcgcttggccgcaatgacctggctgatggtgtgaactcgggccagggcctgggcatcgagatcatcgggaccctccagctggtgctatgcgtgctggctactaccgaccggaggcgccgtgaccttggtggctcagccccccttgccatcggcctctctgtagcccttggacacctcctggctattgactacactggctgtgggattaaccctgctcggtcctttggctccgcggtgatcacacacaacttcagcaaccactggattttctgggtggggccattcatcgggggagccctggctgtactcatctacgacttcatcctggccccacgcagcagtgacctcacagaccgcgtgaaggtgtggaccagcggccaggtggaggagtatgacctggatgccgacgacatcaactccagggtggagatgaagcccaaatag |
| Aqp1_F | TGGACTACAAGGACGACGAC |
| Aqp1_R | CACCTTCACGTTGTCCTGGA |
| MmXBP1s_F | TGAGTCCGCAGCAGGTG |
| MmXBP1s_R | TCCTTCTGGGTAGACCTCTGG |
| MmATF4_F | GAAACCTCATGGGTTCTCCA |
| MmATF4_R | AGAGCTCATCTGGCATGGTT |
| MmCHOP_F | CACCACACCTGAAAGCAGAA |
| MmCHOP_R | GGACGCAGGGTCAAGAGTAG |
| MmBip_F | GGACAAGAAGGAGGATGTGG |
| MmBip_R | TGATCGTTGGCTATGATCTCC |
| MmSYVN1_F | AACCCCACTGAAGAGACTGC |
| MmSYVN1_R | CTCGGGAAGCTCCTCTACAA |
| MmGrp94_F | GTCGGGAAGCAACAGAGAAG |
| MmGrp94_R | TGCCAGACCATCCATACTGA |
| MmGAPDH_F | CCACCCAGAAGACTGTGGAT |
| MmGAPDH_R | CACATTGGGGGTAGGAACAC |
| HsXBP1s_F | GGAGTTAAGACAGCGCTTGGG |
| HsXBP1s_R | CTGCACCTGCTGCGGAC |
| HsATF4_F | TCAAACCTCATGGGTTCTCC |
| HsATF4_R | GTGTCATCCAACGTGGTCAG |
| HsCHOP_F | AGCGACAGAGCCAAAATCA |
| HsCHOP_R | CAGTGTCCCGAAGGAGAAAG |
| HsBip_F | CATGGTTCTCACTAAAATGAAAG |
| HsBip_R | GCTGGTACAGTAACAACTG |
| HsSYVN1_F | CCAACATCTCCTGGCTCTTT |
| HsSYVN1_R | GTCAGGATGCTGTGATAGGC |
| HsGrp94_F | GTCGGAAAAGTTTGCCTTCC |
| HsGrp94_R | TGACATGCAGCAGGTTCTTC |
| HsACTB_F | TTGGCAATGAGCGGTTCC |
| HsACTB_R | GTTGAAGGTAGTTTCGTGGATG |
